# Effectiveness of quality control circle interventions in reducing preoperative anxiety: A cross-sectional observational study

**DOI:** 10.1097/MD.0000000000045037

**Published:** 2026-02-06

**Authors:** Xin Lei, Wei He, Yuqiong Liu, Yuemei Pan, Yulan Xie, Yan Zhu

**Affiliations:** a Department of Stomatology, Shenzhen Longhua District Central Hospital, Shenzhen, Guangdong, China.

**Keywords:** anxiety management, patient satisfaction, perioperative care, postoperative recovery, quality control circle

## Abstract

A widespread problem among surgical patients, preoperative anxiety affects recovery, pain perception and general outcomes and calls for efficient remedies including quality control circle (QCC) approaches to enhance perioperative treatment. This study compared, among surgical patients, the efficacy of QCC treatments in lowering preoperative anxiety, boosting postoperative recovery and raising patient satisfaction against conventional treatment. A cross-sectional observational study was conducted on 180 patients in all who were scheduled for elective operations were allocated to either standard treatment (n = 90) or QCC interventions (n = 90). At admission, 1 day before surgery and 3 days following surgery, anxiety levels were measured with the state-trait anxiety inventory. The numeric rating scale and visual analog scale were used at 2, 12, 24, 48, and 72 hours to gauge postoperative pain. Recovery benchmarks, complications, and satisfaction levels were documented. Preoperatively (*P* = .005) and postoperatively (*P* = .003) patients in QCC treatments group had notably decreased anxiety scores. Recovering times were notably faster (*P* = .001: first drinking, 1.2 vs 3.5 days), first bowel movement (2.1 vs 3.2 days), and off-bed activity (3.0 vs 3.8 days). At all-time-points, the QCC group had regularly lower pain scores (*P* <.01). The QCC group had reduced complications (8.3% vs 15.6% *P* = .021) and greater satisfaction rates (89.4% vs 75.1%, *P* = .005). Ultimately, QCC treatments minimized the consequences by efficiently lowering anxiety, raising recovery benchmarks, improving pain management and raising patient satisfaction.

## 1. Introduction

Preoperative anxiety is a frequent psychological response in patients awaiting surgery, characterized by heightened worry, fear, and physiological stress in anticipation of surgical outcomes and hospitalization.^[1,2]^ This anxiety can adversely impact both mental and physical health, leading to increased perioperative stress, heightened pain perception, and delayed postoperative recovery.^[3]^ Therefore, effective management of preoperative anxiety has become an essential component of modern perioperative care.

Quality control circles (QCCs) are collaborative, small-group interventions rooted in continuous quality improvement principles. Originally developed for industrial quality assurance, QCCs have been successfully adapted in healthcare settings to address clinical workflow inefficiencies and improve patient care outcomes.^[4,5]^ A typical QCC consists of a group of healthcare professionals – often nurses or interdisciplinary staff – who voluntarily meet to identify problems, analyze root causes, and implement targeted interventions.

In healthcare, QCCs have been applied to improve a variety of outcomes. For example, they have been shown to reduce postoperative infection rates,^[5]^ enhance adherence to clinical safety protocols,^[6]^ and accelerate recovery milestones such as earlier ambulation and shorter hospital stays.^[7]^ These findings demonstrate the potential of QCCs to improve both patient safety and care efficiency. However, the existing evidence base is limited in both scope and methodological rigor. Most QCC studies in healthcare have been descriptive, single-center, or process-oriented in design, with limited use of validated outcome measures and minimal attention to patient-centered psychological endpoints.^[8,9]^

Beyond direct patient education and relaxation strategies, QCCs are designed to strengthen staff responsibility, communication, and teamwork. These factors are theoretically linked to patient outcomes, as cohesive and communicative care teams provide more consistent emotional support, clearer information, and greater responsiveness to patient concerns – all of which contribute to reduced preoperative anxiety and improved perioperative experiences.^[6]^ Measuring such “invisible achievements” among staff therefore complements patient-centered outcomes and clarifies the mechanisms through which QCCs exert their effects.

Only a few scattered reports have hinted that QCC frameworks may reduce preoperative anxiety, but these studies lacked rigorous methodology, used heterogeneous outcome measures, and did not apply validated psychometric tools such as the State-Trait Anxiety Inventory (STAI). Consequently, it remains unclear whether QCC interventions can reliably improve perioperative psychological outcomes, in addition to their demonstrated benefits on clinical efficiency.^[10,11]^

While elements such as counseling and relaxation are individually established in perioperative care, the distinguishing feature of QCC is its structured, team-based quality improvement framework. Through iterative PDCA (Plan–Do–Check–Act) cycles, frontline staff collaboratively identify patient needs, select targeted strategies, and refine their delivery, ensuring that interventions are both context-specific and continuously optimized. This makes QCC distinct from conventional multimodal protocols, which apply standardized interventions without systematic feedback-driven adaptation.^[11,12]^

The present study was therefore designed to address this knowledge gap by assessing the efficacy of a bundled QCC intervention in reducing preoperative anxiety and improving perioperative outcomes. By focusing on validated psychological measures (STAI), postoperative recovery benchmarks, complications, and patient satisfaction, this study provides new evidence on the potential of QCC as an organizational framework for improving patient-centered perioperative care.

## 2. Materials and methods

This cross-sectional observational study was conducted at Shenzhen Longhua District Central Hospital, Shenzhen, China, from February 2023 to September 2024, to assess the efficacy of QCC interventions in alleviating preoperative anxiety in patients undergoing elective procedures. A convenience sampling method was employed to enroll 180 participants who met the inclusion criteria. This non-probability approach was selected primarily due to logistical and practical constraints, including limited time, resource availability, and patient flow within the study setting. Given the clinical environment and the need to implement interventions in a timely manner, convenience sampling enabled efficient recruitment while maintaining clinical relevance.

**Figure 1.**
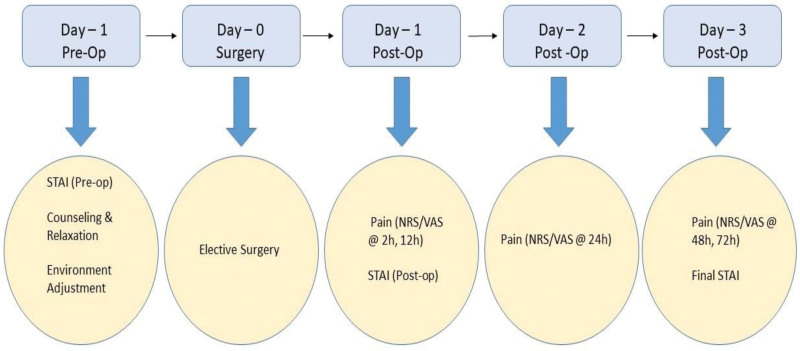
Timeline of QCC interventions and assessment schedule showing key time-points for STAI (anxiety), NRS/VAS (pain) and recovery evaluations. QCC = quality control circle, NRS = NRS = numeric rating scale, SAI = state anxiety inventory, STAI = state-trait anxiety inventory, TAI = trait anxiety inventory, VAS = visual analog scale.

Participants were assigned to either the QCC intervention group (n = 90) or the standard care group (n = 90) based on their date of admission and operating room scheduling to avoid selection bias from clinician assignment (Fig. 1). Although randomization was not performed, the groups were checked for baseline comparability on key demographic and clinical characteristics (e.g., age, gender, ASA classification, BMI), and no statistically significant differences were found. Stratified group allocation within the sampling constraints helped minimize allocation imbalance.

## 3. Participants and procedures

Participants aged 18 to 65 years scheduled for intermediate-complexity elective surgery under general anesthesia were eligible if they could comprehend and respond to questionnaires. Eligible procedures included laparoscopic cholecystectomy, appendectomy, gynecological laparoscopic surgeries (such as ovarian cystectomy and myomectomy), and minor orthopedic operations. These procedures were selected because they are routine at our institution, relatively comparable in invasiveness and recovery trajectory, and represent surgical contexts where perioperative anxiety is clinically relevant.

To avoid bias introduced by extremes of surgical complexity, patients undergoing high-complexity procedures (e.g., oncologic resections, cardiothoracic surgery, or organ transplantation) and those undergoing very minor day-case procedures (e.g., superficial soft-tissue excisions) were excluded. This ensured a relatively homogeneous surgical cohort in terms of anticipated physiological stress, pain profiles, and recovery patterns.

Participants were excluded if they were undergoing emergency operations, had a diagnosed mental illness, systemic disease, coagulation disorder, or a history of multiple prior surgeries.

Preoperative physical health status was evaluated using the American Society of Anesthesiologists (ASA) Physical Status Classification System, where ASA I denotes a normal healthy patient and ASA II indicates a patient with mild systemic disease that does not limit physical activity. Only ASA I and ASA II patients were included to ensure relative homogeneity in baseline surgical risk.

To standardize surgical conditions, we restricted inclusion to procedures of similar invasiveness and operative complexity. Patients undergoing high-complexity oncologic, cardiothoracic, or transplant procedures, as well as minor day-case operations (e.g., superficial soft-tissue excision), were excluded. This ensured that both groups (QCC and standard care) had broadly “uniform” surgical conditions, thereby minimizing confounding effects related to procedure type or severity.

Ethical approval was obtained from the Institutional Ethics Committee (SZLHQ/2713, dated January 9, 2023), and written informed consent was obtained from all participants.

Importantly, the distribution of surgical procedure types was comparable between the QCC intervention and standard care groups, with no significant differences in invasiveness, duration, or complexity. Consistency was further confirmed by analysis of operative duration and intraoperative blood loss, which showed no statistically significant differences (Table 1).

**Table 1:** Postoperative recovery outcomes.

Parameter	QCC interventions (n = 90)	Standard care (n = 90)	*t*	*P*-value
Operation time (min)	230.4 ± 45.2	228.6 ± 42.8	0.31	.757
Intraoperative blood loss (mL)	350.1 ± 85.7	340.3 ± 79.5	0.54	.594
First drinking time (days)	1.2 ± 0.5	3.5 ± 0.8	15.42	<.001
First exhaust time (days)	1.8 ± 0.6	3.0 ± 0.9	10.73	<.001
First bowel movement time (days)	2.1 ± 0.7	3.2 ± 1.0	8.41	<.001
First off-bed activity (days)	3.0 ± 0.8	3.8 ± 0.9	5.93	<.001

## 4. Assessment tools

Anxiety was assessed using the STAI, Form Y, comprising:

1. SAI (State Anxiety Inventory – Form Y-1): Measures current, situational anxiety.
2. TAI (Trait Anxiety Inventory – Form Y-2): Assesses general anxiety tendencies.

The Chinese (Mandarin) version, previously validated for use in surgical populations, was administered at 3 time-points: upon admission, 1 day before surgery, and 3 days postoperatively.

Postoperative pain was measured using:

1. Numeric Rating Scale (NRS) and
2. Visual Analog Scale (VAS)

Both at 5 intervals: 2, 12, 24, 48, and 72 hours postoperatively. Patient satisfaction was evaluated using a 5-point Likert-scale survey, though it is local validation was not formally confirmed. Postoperative complications (e.g., nausea, vomiting, wound infection, respiratory issues) were defined based on clinical criteria and recorded through standardized chart reviews and clinician reports.

In addition to clinical and psychological outcomes, the study also assessed team-related “invisible outcomes” such as sense of responsibility, joviality, communication, and teamwork. These were evaluated using a structured 10-point Likert-scale-based questionnaire developed for internal hospital quality improvement projects. Each item ranged from 1 (lowest) to 10 (highest), and higher scores reflected stronger agreement or more positive perceptions.

## 5. QCC intervention

The QCC intervention was delivered by a team of 10 nurses with 5 to 10 years of clinical experience. The intervention included:

- Individualized counseling sessions: Two 30-minute sessions (1 at admission and 1 a day before surgery) covering procedure education, breathing exercises, and addressing patient concerns.
- Environmental modifications: Adjustments to ambient temperature, quiet zones, and individualized waiting area setups.
- Relaxation techniques: Guided breathing and audio-based calming sessions.

To ensure fidelity, the QCC team followed SOPs and completed checklist-based intervention logs for each patient. Each QCC nurse was trained using a standardized guide specific to perioperative anxiety management. A senior nurse supervisor conducted random audits of 20% of cases and offered real-time feedback during weekly QCC meetings. All QCC nurses participated in a structured 2-day training workshop that included didactic sessions on perioperative anxiety, role-play of counseling interactions, and supervised practice of relaxation guidance. A standardized intervention manual and SOP-based checklists ensured consistency across cases. Fidelity was maintained through patient-specific logs completed after each session, and a senior nurse supervisor randomly audited 20% of cases, providing real-time feedback during weekly QCC review meetings. While the 3 core intervention elements (counseling, relaxation, environmental modification) were delivered uniformly, minor tailoring was permitted to accommodate individual patient concerns within the standardized framework.

To avoid contamination, QCC elements were strictly limited to the intervention group. Standard care patients received routine group educational sessions and preoperative information leaflets, but no individualized counseling or structured feedback mechanisms.

The QCC protocol was delivered as a structured, bundled intervention, reflecting how QCCs are implemented in real-world hospital practice. Similar to ERAS protocols, the effectiveness of QCCs stems not from a single element but from the interaction of multiple targeted strategies. Therefore, our design prioritized clinical applicability over disaggregated mechanistic testing.

## 6. Statistical analysis

All analyses were performed using SPSS version 26.0 (Chicago). Continuous variables are presented as mean ± standard deviation, and categorical variables as frequencies or percentages. Baseline demographic and operative characteristics were compared between groups using independent-sample t-tests for continuous variables and Chi-square tests for categorical variables.

For longitudinal outcomes (STAI anxiety scores measured at admission, 1 day before surgery, and 3 days postoperatively), a repeated-measures ANOVA was used, with group (QCC vs standard care) as the between-subjects factor and time as the within-subjects factor. When sphericity assumptions were violated, Greenhouse–Geisser corrections were applied. post hoc pairwise comparisons were adjusted using Bonferroni correction.

A *P*-value of <.05 was considered statistically significant.

All 180 enrolled participants completed the study, and there were no dropouts or losses to follow-up. Complete case analysis was conducted for all variables.

## 7. Results

The outcomes of postoperative recovery for the standard care and QCC groups indicated that patients were exposed to comparable surgical conditions, with no statistically significant differences in operation duration (*P* = .757) or intraoperative blood loss (*P* = .594). Despite this baseline equivalence, patients in the QCC group demonstrated markedly faster recovery milestones. The time to first oral intake was significantly shorter in the QCC group (1.2 ± 0.5 vs 3.5 ± 0.8 days, *P* <.001), and the time to first off-bed activity was also earlier (3.0 ± 0.8 vs 3.8 ± 0.9 days, *P* <.001). These findings suggest that QCC interventions may facilitate more rapid restoration of gastrointestinal function and early mobilization, both of which are considered critical indicators of enhanced recovery after surgery (ERAS). Importantly, these improvements were achieved without prolonging operative time or increasing intraoperative blood loss, further supporting the potential of QCC protocols to improve postoperative recovery efficiency (Table 1).

**Figure 2.**
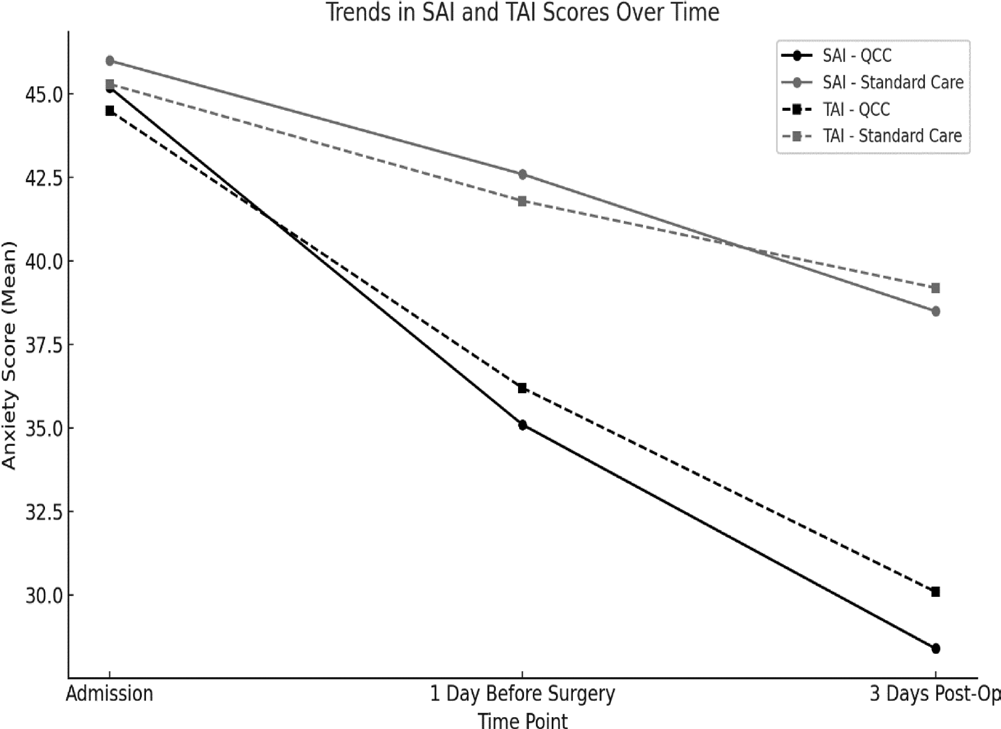
Trends in SAI and TAI scores over time. QCC interventions significantly reduced anxiety levels from admission to postoperative day 3 compared to standard care. QCC = quality control circle, SAI = state anxiety inventory, TAI = trait anxiety inventory.

At admission, no significant differences were observed between groups in SAI (QCC: 45.2 ± 8.5, Standard Care: 46.0 ± 8.3, *P* = .621) or TAI (QCC: 44.5 ± 8.7, Standard Care: 45.3 ± 8.5, *P* = .687). However, by 1 day before surgery, the QCC group had significantly lower SAI (35.1 ± 7.4) and TAI (36.2 ± 7.8) scores compared to standard care (SAI: 42.6 ± 7.9, TAI: 41.8 ± 8.3, *P* <.001 for both). This trend persisted at 3 days post-op (SAI: 28.4 ± 6.8 vs 38.5 ± 7.2, TAI: 30.1 ± 7.1 vs 39.2 ± 7.9, *P* <.001) (Table 2) and the overall trajectory of anxiety reduction illustrated in Figure 2.

**Table 2:** Comparison of state and trait anxiety levels over time between groups.

Time point	SAI – QCC interventions (mean ± SD)	SAI – standard care (mean ± SD)	TAI – QCC interventions (mean ± SD)	TAI – standard care (mean ± SD)	Min–max range	Median (IQR) QCC/std care	Variance QCC/std care	Std. error QCC/std care	*P*-value (SAI)	*P*-value (TAI)
Admission	45.2 ± 8.5	46.0 ± 8.3	44.5 ± 8.7	45.3 ± 8.5	20–60	28 (25–31)/ 38 (34–42)	72.25/ 78.14	1.21/ 1.34	.621	.687
1 d before surgery	35.1 ± 7.4	42.6 ± 7.9	36.2 ± 7.8	41.8 ± 8.3	20–60	28 (25–31)/38 (34–42)	72.25/78.14	1.21/1.34	<.001	<.001
3 d postoperative	28.4 ± 6.8	38.5 ± 7.2	30.1 ± 7.1	39.2 ± 7.9	20–60	28 (25–31)/38 (34–42)	72.25/78.14	1.21/1.34	<.001	<.001

QCC = quality control circle, SAI = state anxiety inventory, SD = standard deviation, TAI = trait anxiety inventory.

Postoperative pain assessed via NRS and VAS at multiple time-points showed consistent differences. At 2 hours, the QCC group reported NRS 4.5 ± 1.1 and VAS 4.99 ± 0.94, compared to 6.2 ± 1.3 and 6.26 ± 1.20 in the standard care group (*P* = .001 for both). Significant differences remained at 12, 24, and 48 hours (*P* <.01). At 72 hours, NRS remained significantly different (*P* = .007), while VAS differences were not statistically significant (*P* = .285) (Table 3).

**Table 3:** Postoperative pain levels over time (NRS and VAS).

Time point	NRS – QCC interventions (mean ± SD)	NRS – standard care (mean ± SD)	VAS – QCC Interventions (Mean ± SD)	VAS – standard care (mean ± SD)	Min–max range	Median (IQR)	Variance	Std. error	*P*-value (NRS)	*P*-value (VAS)
2 h	4.5 ± 1.1	6.2 ± 1.3	4.99 ± 0.94	6.26 ± 1.20	1–6/3–8	2 (2–3)/ 4 (3–5)	1.21/ 1.69	0.15/ 0.22	.001	<.001
12 h	4.1 ± 1.0	5.7 ± 1.1	3.20 ± 0.85	5.59 ± 1.03	1–6/3–8	2 (2–3)/ 4 (3–5)	1.21/ 1.69	0.15/ 0.22	.002	<.001
24 h	3.2 ± 0.9	5.1 ± 1.0	2.50 ± 0.57	5.08 ± 0.93	1–6/3–8	2 (2–3)/ 4 (3–5)	1.21/ 1.69	0.15/ 0.22	.003	<.001
48 h	2.5 ± 0.8	4.3 ± 0.9	1.89 ± 0.47	2.00 ± 0.59	1–6/3–8	2 (2–3)/ 4 (3–5)	1.21/ 1.69	0.15/ 0.22	.005	.267
72 h	1.8 ± 0.7	3.5 ± 0.8	1.78 ± 0.29	1.85 ± 0.36	1–6/3–8	2 (2–3)/ 4 (3–5)	1.21/ 1.69	0.15/ 0.22	.007	.285

NRS = numeric rating scale, QCC = quality control circle, SD = standard deviation, VAS = visual analog scale.

Regarding the comparison between groups of QCC interventions and standard care, patient satisfaction and complication rates, the overall satisfaction level of 89.4 ± 5.2% the QCC interventions group showed far more than the standard care group (75.1 ± 6.1%, *P* = .005). Moreover, QCC interventions group (82.3%) had clearly larger percentage of highly satisfied patients than standard care group (65.4%, *P* = .004). On the other hand, less patients in QCC interventions group said they were either unhappy (5.2% vs 9.9%, *P* = .004) or somewhat satisfied (12.5% vs 24.7%, *P* = .004), so demonstrating that QCC interventions successfully changed patient experiences and views of treatment. With regard to complication rates, QCC interventions group displayed much lower incidence of complications (8.3% vs 15.6%, *P* = .021), implying that the organized strategies used by the QCC team help to improve clinical results and lessen postoperative risks (Table 4).

**Table 4:** Patient satisfaction and complications.

Parameter	QCC interventions (n = 90)	Standard care (n = 90)	Min–max range	Median (IQR)	Variance	Std. error	*P*-value
Satisfaction (%)	89.4 ± 5.2	75.1 ± 6.1	75–95	88 (85–91)	9.12	1.03	.005
Highly satisfied (%)	82.3	65.4	–	–	–	–	.004
Moderately satisfied (%)	12.5	24.7	–	–	–	–	.004
Dissatisfied (%)	5.2	9.9	–	–	–	–	.004
Complication rate (%)	8.3	15.6	5–18	8 (7–10)	5.45	0.78	.021

QCC = quality control circle.

With no statistically significant difference (*P* = .62), the age distribution was somewhat similar between the standard care group (46.2 ± 11.8 years) and QCC Interventions group (45.6 ± 12.3 years). Comparable were the gender distribution (48/42 vs 50/40, *P* = .78) and BMI values (24.7 ± 3.2 vs 25.1 ± 3.4, *P* = .53), therefore guaranteeing consistency across important demographic factors. Indicating similar preoperative health conditions in both groups, ASA classification that of ASA’ physical status was likewise statistically similar (*P* = .48). These results confirmed that the QCC interventions, not demographic or baseline variations, accounted for any noted variations in results (Table 5).

**Table 5:** Demographics and baseline characteristics.

Characteristic	QCC interventions (n = 90)	Standard care (n = 90)	*P*-value
Age (year)	45.6 ± 12.3	46.2 ± 11.8	.62
Gender (male/female)	48/42	50/40	.78
BMI (kg/m^2^)	24.7 ± 3.2	25.1 ± 3.4	.53
ASA classification I/II (%)	55/45	52/48	.48

ASA = American Society of Anesthesiologists, BMI = body mass index, QCC = quality control circle.

The invisible successes among staff members and patients who had undergone QCC interventions, with regard to all measured criteria, QCC interventions group scored noticeably better than the standard care group (*P* = .001). Professional knowledge (7.63 ± 0.92) sense of responsibility and honor (9.36 ± 0.50 vs 4.82 ± 0.75), and self-confidence (8.55 ± 0.69 vs 5.09 ± 0.83) showed particular improvement. Better team cohesion (8.73 ± 1.01 vs 5.18 ± 0.87), communication (8.09 ± 1.04 vs 4.73 ± 0.91), and cooperation (9.18 ± 0.75 vs 4.91 ± 1.04) the QCC group also shown better than higher degrees of joviality (7.63 ± 0.92) were also recorded by participants compared to 4.72 ± 0.78. These findings underlined how well QCC treatments promoted teamwork, staff engagement and interpersonal communication, hence boosting staff performance and patient outcomes (Table 6).

**Table 6:** Invisible achievement scores before and after QCC.

Parameter	QCC interventions (mean ± SD)	Standard care (mean ± SD)	*t*	*P*-value
Professional knowledge	7.63 ± 0.92	4.72 ± 0.78	7.95	<.001
Sense of responsibility and honor	9.36 ± 0.50	4.82 ± 0.75	16.67	<.001
Self-confidence	8.55 ± 0.69	5.09 ± 0.83	10.62	<.001
Team cohesion	8.73 ± 1.01	5.18 ± 0.87	8.81	<.001
Communication	8.09 ± 1.04	4.73 ± 0.91	8.07	<.001
Cooperation	9.18 ± 0.75	4.91 ± 1.04	11.02	<.001
Sense of joviality	7.63 ± 0.92	4.72 ± 0.78	7.95	<.001

QCC = quality control circle, SD = standard deviation.

**Figure 3.**
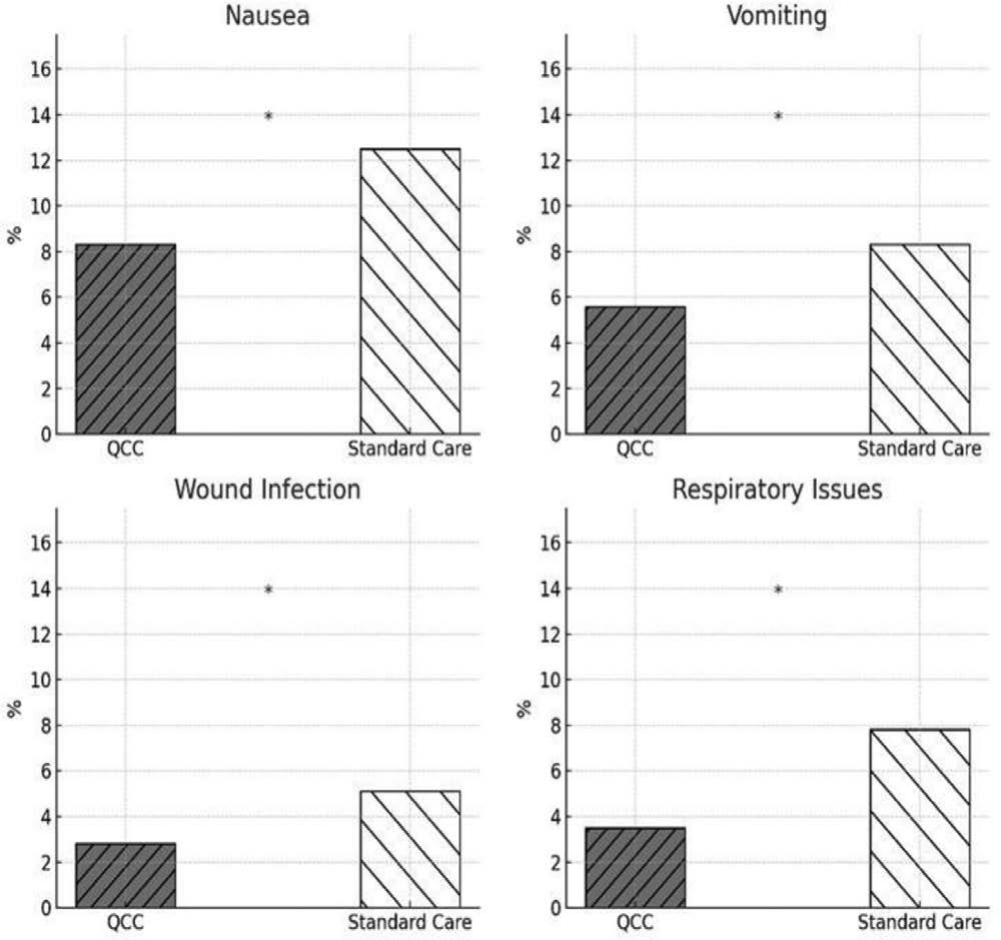
Complications and adverse events.

Comparing the incidence of postoperative complications, nausea, vomiting, wound infection and respiratory problems, between the QCC interventions and standard care groups, the graphs showed the QCC interventions group indicated reduced complication rates than the standard care group. The QCC group showed much lower nausea (8% vs 13%) and vomiting (6% vs 8%), which would represent improved postoperative treatment. Comparatively, events of respiratory problems (2% vs 6%) and wound infection (3% vs 5%) were much fewer, underlining the success of QCC treatments in lowering postoperative complications and enhancing patient outcomes (Fig. 3).

**Figure 4.**
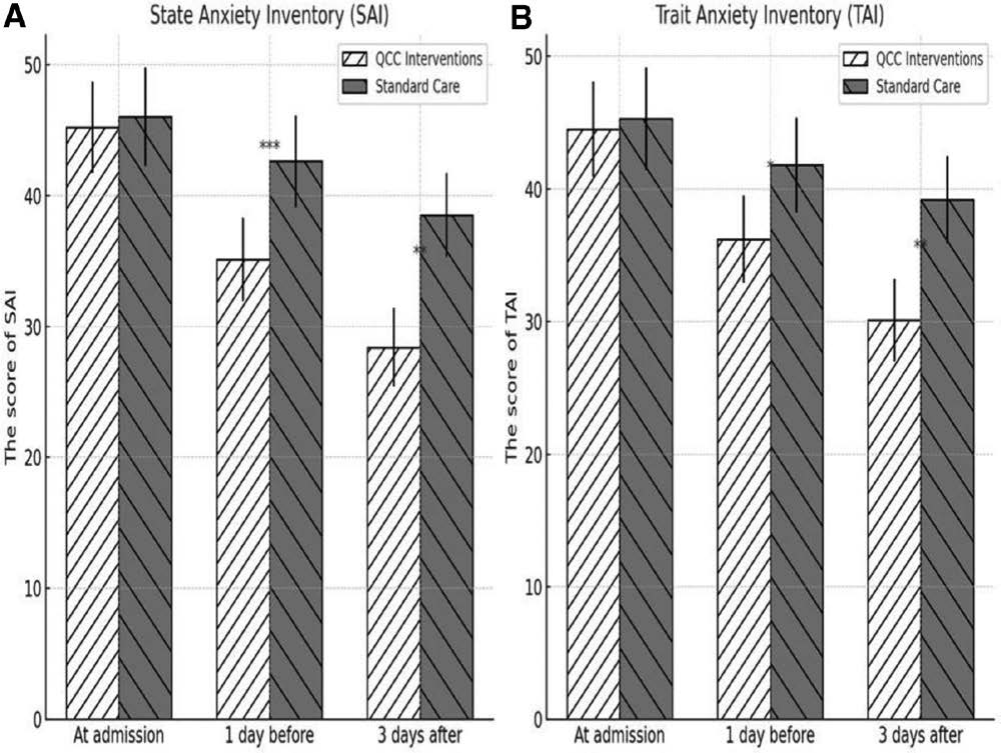
Comparison of anxiety inventory scores between QCC interventions and standard care. (A) SAI. (B) TAI. QCC = quality control circle, SAI = state anxiety inventory, TAI = trait anxiety inventory.

Over time, the graphs showed TAI and SAI scores among groups between QCC interventions and standard care. Although both groups had equal admission scores, QCC interventions group showed noticeably decreased anxiety levels 3 days after surgery and 1 day before (*P* <.001 and *P* <.05), therefore showing that QCC interventions successfully lowered preoperative and postsurgical anxiety (Fig. 4).

## 8. Discussion

### 8.1. Anxiety reduction and psychological impact

This study demonstrated a significant reduction in both state and trait anxiety scores in the QCC intervention group compared to standard care, particularly on the day before surgery and 3 days postoperatively. While these outcomes suggest beneficial effects of QCC, they should be interpreted with caution and nonspecific factors such as increased staff–patient interaction or heightened perception of care quality may also have contributed. These results align with recent findings from Gu et al. (2023), which reported decreased anxiety levels in patients who received personalized counseling and relaxation training before laparoscopic gynecological surgery.^[13]^ The integration of individualized education, guided breathing exercises, and environmental adjustments in our QCC protocol contributed to this reduction. Other recent studies reinforce the value of multimodal non-pharmacologic interventions in perioperative anxiety management, highlighting that such strategies yield psychological benefits beyond what medication alone offers.^[8,9]^ These findings should not be interpreted as replication of conventional multimodal anxiety management. While the individual techniques (counseling, relaxation, environmental modification) are well established, QCC provides a structured, collaborative, and feedback-driven framework for integrating and continuously refining these elements. This theoretical distinction highlights QCC as an organizational model rather than a simple aggregation of interventions.

Traditional pharmacologic approaches like benzodiazepine premedication have been criticized for providing only transient relief and for causing side effects such as cognitive dulling or dependency.^[11,12]^ The QCC model’s collaborative and patient-centered framework directly addressed emotional concerns and created a sense of preparedness, which likely contributed to improved anxiety outcomes. This confirms earlier conclusions by Mulugeta et al. (2018), who emphasized the need for patient-specific engagement to reduce psychological distress.^[14]^

### 8.2. Postoperative recovery milestones

The QCC group experienced significantly faster recovery benchmarks, including earlier first drinking (1.2 vs 3.5 days), bowel movement, and off-bed activity. These improvements are consistent with the findings of Li et al. (2024), who showed that structured nursing interventions focusing on education and psychological readiness can accelerate gastrointestinal and mobility recovery.^[15]^ Reduced anxiety may indirectly enhance physiological recovery by minimizing stress-induced suppression of digestive and immune functions, as supported by Beukeboom et al. (2012).^[16]^

### 8.3. Pain management

Postoperative pain levels, measured using NRS and VAS, were consistently lower in the QCC group across nearly all-time-points. The most notable differences were observed within the first 48 hours post-surgery. These findings support the hypothesis that psychological readiness and emotional support can modulate pain perception. Farrar et al. (2010) observed similar patterns, where patients receiving preoperative psychosocial support reported lower pain intensity and better medication responses.^[17]^ Additionally, Woo (2010) posited that lowered anxiety helps attenuate central pain processing mechanisms, a finding that supports the use of QCC interventions as indirect pain modulators.^[18]^

### 8.4. Clinical complications and patient satisfaction

A significant reduction in postoperative complications was observed in the QCC group (8.3% vs 15.6%, *P* = .021). Complications such as nausea, vomiting, wound infections, and respiratory issues were less frequent, possibly due to early mobilization, improved patient understanding, and reduced perioperative stress. These findings echo those of Shi and Zhang (2021), who demonstrated lower infection rates in orthopedic surgery patients exposed to QCC protocols.^[19,20]^

Patient satisfaction was also notably higher (89.4% vs 75.1%, *P* = .005), consistent with the results of Pereira et al. (2016), who reported that patient-centered care models substantially improve satisfaction by addressing emotional and informational needs.^[12]^

### 8.5. Staff outcomes and team performance

In addition to patient outcomes, the study also captured the “invisible achievements” among nursing staff. Members of the QCC group reported significantly higher levels of professional confidence, communication, teamwork, and overall morale. These findings are consistent with more recent literature emphasizing the benefits of interdisciplinary collaboration on clinical efficacy and workplace satisfaction.^[21]^ Enhanced team cohesion and open communication channels not only improve internal workflow but also translate into better patient care.

### 8.6. Clinical implications

The current findings affirm that QCC interventions are effective not only in reducing perioperative anxiety but also in improving recovery, pain control, patient satisfaction, and team performance. By aligning with modern patient-centered care principles, QCCs address both physical and psychological aspects of surgical treatment.

## 9. Limitations

This study has several important limitations. First, the design did not isolate the individual contributions of counseling, environmental modifications, and relaxation training. As with ERAS protocols, however, QCCs are typically delivered as bundled interventions in clinical practice, and their effectiveness arises from the synergy between multiple elements. Our pragmatic design deliberately preserved this integrated framework but precludes identification of which specific components drove the observed effects.

Second, the use of non-randomized convenience sampling with allocation based on admission scheduling may have introduced selection bias. Although baseline demographic and clinical characteristics were statistically comparable between groups, residual confounding from unmeasured factors cannot be excluded. Accordingly, the findings should be interpreted as associational rather than causal evidence. Future randomized trials or analytic approaches such as propensity score matching are needed to strengthen causal inference.

Third, while the QCC protocol was delivered using standardized training, manuals, and checklist-based fidelity monitoring, limited tailoring was permitted to address individual patient concerns (e.g., counseling emphasis or environmental adjustments). Although this introduces some heterogeneity, it reflects the pragmatic, patient-centered adaptability of real-world perioperative care. Importantly, all tailoring occurred within predefined boundaries, ensuring that the intervention remained structured and comparable across participants.

Finally, the external validity of the findings is restricted by the single-center design, reliance on convenience-based sampling, and focus on ASA I–II patients undergoing intermediate-complexity elective procedures. Results may not be generalizable to patients undergoing high-complexity oncologic or cardiothoracic operations, emergency surgeries, or to institutions with different staffing structures, patient demographics, or perioperative workflows. Future multicenter studies with broader surgical populations are required to enhance generalizability and establish broader applicability.

## 10. Conclusion

QCC interventions demonstrated meaningful improvements in perioperative care, including reductions in patient anxiety, faster recovery milestones, lower postoperative pain, and higher satisfaction. These results highlight the value of QCCs as a structured, team-based framework that can be integrated into routine surgical workflows with minimal additional resources, while also strengthening staff collaboration. Although the present findings provide pragmatic evidence of effectiveness, they should be interpreted as associational rather than causal. Future multicenter, randomized studies with long-term follow-up and advanced analytical methods are needed to validate these outcomes, clarify active intervention components, and support broader adoption of QCCs in perioperative protocols.

## Author contributions

**Conceptualization:** Wei He, Yuemei Pan, Yan Zhu.

**Data curation:** Wei He, Yuemei Pan.

**Formal analysis:** Yuemei Pan.

**Investigation:** Yuqiong Liu, Yulan Xie, Yan Zhu.

**Methodology:** Xin Lei, Yuqiong Liu, Yulan Xie, Yan Zhu.

**Writing – review & editing:** Xin Lei, Yan Zhu.
